# Ginsenoside Rb1 increases insulin sensitivity by activating AMP-activated protein kinase in male rats

**DOI:** 10.14814/phy2.12543

**Published:** 2015-09-09

**Authors:** Ling Shen, Michael Haas, David Q-H Wang, Aaron May, Chunmin C Lo, Silvana Obici, Patrick Tso, Stephen C Woods, Min Liu

**Affiliations:** 1 Department of Pathology and Laboratory Medicine, University of Cincinnati College of MedicineCincinnati, Ohio; 2 Department of Cancer & Cell Biology, University of Cincinnati College of MedicineCincinnati, Ohio; 3 Department of Internal Medicine, Saint Louis University School of MedicineSt. Louis, Missouri; 4 Department of Internal Medicine, University of Cincinnati College of MedicineCincinnati, Ohio; 5 Department of Psychiatry and Behavioral Neuroscience, University of Cincinnati College of MedicineCincinnati, Ohio

**Keywords:** AMPK, ginsenoside, glucose homeostasis, insulin signaling pathway, TBC1D4

## Abstract

Although ginseng has been reported to ameliorate hyperglycemia in animal models and clinical studies, the molecular mechanisms are largely unknown. We previously reported that chronic treatment with ginsenoside Rb1 (Rb1), a major component of ginseng, significantly reduced fasting glucose and improved glucose tolerance in high-fat diet (HFD)-induced obese rats. These effects were greater than those observed in pair-fed rats, suggesting a direct effect of Rb1 on glucose homeostasis, and this possibility was confirmed in the present study. In lean rats fed standard rodent chow, 5-day treatment with Rb1 significantly improved glucose tolerance and enhanced insulin sensitivity. Notably, those effects were not accompanied by reduced food intake or changed body weight. To elucidate the underlying molecular mechanisms, rats fed a HFD for 4 weeks were treated with Rb1 for 5 days. Subsequently, euglycemic-hyperinsulinemic clamp studies found that compared to vehicle, Rb1, while not changing food intake or body weight, significantly increased glucose infusion rate required to maintain euglycemia. Consistent with this, insulin-induced inhibition of hepatic gluconeogenesis was significantly enhanced and hepatic phosphoenolpyruvate carboxykinase and glucose-6-phosphatase gene expression was suppressed. Additionally, glucose uptake was significantly increased in skeletal muscle. While proximal insulin signaling was not changed after Rb1 treatment, increased phosphorylation of TBC1D4, a downstream target of AMPK signaling, appears to be a key part of the mechanism for Rb1-stimulated glucose uptake in skeletal muscle. These findings indicate that Rb1 has multiple effects on glucose homeostasis, and provide strong rationale for further evaluation of its potential therapeutic role.

## Introduction

The prevalence of the metabolic syndrome and type-2 diabetes (T2DM) has risen enormously in both developed and developing countries over the last decades (Lam and LeRoith 2012). Despite advances in treatment, the goals of long-term diabetes management generally remain unmet (Levy 2009). At the same time, interest in complementary and alternative medicine continues to grow, becoming an important therapeutic approach sought by some individuals with diabetes (Chang et al. 2007; DiNardo et al. 2012). Ginseng is one of the most popular and best-selling herbs in the United States (Blumenthal et al. 2006), and the most extensively studied alternative medicine for treatment of T2DM (Franz et al. 2003). Ginseng has been reported to ameliorate hyperglycemia in animal (Kimura et al. 1981; Attele et al. 2002; Franz et al. 2003; Yun et al. 2004) and human studies (Sotaniemi et al. 1995; Vuksan et al. 2000a, 2001b). In rodents, an extract of ginseng root prevented weight gain and lowered fasting glucose and triglyceride levels in high-fat diet (HFD)-induced obese mice (Yun et al. 2004). Chronic oral administration of ginseng extract also counteracted the effects of high-fructose-induced insulin resistance in rats (Liu et al. 2005). Interestingly, treatment with a ginseng extract significantly reduces fasting blood glucose and stabilizes postprandial glycemia in both noninsulin-dependent diabetic patients and healthy subjects (Sotaniemi et al. 1995; Vuksan et al. 2000b), suggesting that ginseng may also benefit healthy individuals. Ginseng treatment also reduces T2DM-associated risk factors (e.g., hyperlipidemia and hypertension), and improves psychophysical performance (Sotaniemi et al. 1995; Vuksan et al. 2001b). These reports collectively suggest that some constituent(s) in the ginseng extract are important in ameliorating obesity and diabetes.

Pharmacological studies of ginseng have a long history, and most of the pharmacological actions of ginseng are attributed to a family of compounds called ginsenosides (Huang 1999). Ginsenoside Rb1 (Rb1), the most abundant and likely the most important component of the ginsenosides (Washida and Kitanaka 2003), has diverse biological activities. It facilitates acquisition and retrieval of memory, and enhances cognitive function in animal models, perhaps via potentiating hippocampal cholinergic activity (Zhang et al. 1990; Mook-Jung et al. 2001). Intra-third-ventricular injection of Rb1 dose-dependently decreases meal size with no change of intermeal interval (Etou et al. 1988). Our previous studies demonstrated that chronic administration of Rb1 significantly reduced fasting plasma glucose and improved impaired glucose tolerance in HFD-induced obese rats without increasing plasma insulin, indicating that Rb1 is efficacious in ameliorating diabetic symptoms associated with HFD-induced obesity (Xiong et al. 2010). Since the magnitude of the improvements were greater than those observed in pair-fed controls (Xiong et al. 2010), the implication is that while Rb1’s antihyperglycemic action can be partially attributed to reduced food intake and body weight, there are likely direct effects of Rb1 on glucose homeostasis as well. The goals of the current study were (1) to determine whether Rb1 treatment could improve glucose handling in lean rats without significant change in body weight, and (2) to elucidate Rb1’s underlying molecular mechanisms using the euglycemic-hyperinsulinemic clamp, the “gold standard” for assessing insulin action in vivo, in HFD-fed rats.

## Methods

### Animals and diets

Adult male Long–Evans rats (Harlan, Indianapolis, IN) were individually housed in a temperature-controlled vivarium on a 12/12 h light/dark cycle (lights on at 0400 h). Lean rats were maintained on standard rat chow (Purina, St. Louis, MO). Low-fat diet (LFD, category number D03082705) or HFD (category number D03082706) produced by Research Diets, Inc. (New Brunswick, NJ) (Woods et al. 2003) were provided ad libitum to rats for the hyperinsulinemic clamp study. All animal procedures were approved by the Institutional Animal Care and Use Committee of the University of Cincinnati.

### Materials

Rb1 purified from ginseng roots by high-performance liquid chromatography (HPLC) was purchased from Jilin University in China. HPLC (Shimadzu, Kyoto, Japan) analysis performed in our laboratory confirmed that the Rb1 had a purity of ≥98% using an Rb1 standard obtained from LKT laboratories (St. Paul, MN) (Xiong et al. 2010). d-[3-^3^H]glucose and 2-[^14^C]DG (2-deoxy-d-glucose) were purchased from PerkinElmer, Inc. (Waltham, MA). Catheters (Micro-Renathane MRE-033) were purchased from Braintree Scientific. Insulin (Humulin R) was from Eli Lilly (Indianapolis, IN), and 50% dextrose solution was from Henry Schein (Melville, NY). Antibodies for phosphorylated AMP-activated protein kinase *α* (pAMPK*α*) (Thr^172^) and AMPK*α*, phospho-acetyl-CoA carboxylase (pACC) and ACC, GLUT4, IRS-1, phospho-Akt (pAkt), and Akt, phospho-TBC1D4 (pTBC1D4) and TBC1D4, were purchased from Cell Signaling Technology (Danvers, MA). Antibody for phospho-insulin receptor substrate 1 (pIRS-1) was purchased from Santa Cruz Biotechnology, Inc. (Dallas, TX), and antibody for actin was from EMD Millipore (Billerica, MA).

### Intraperitoneal glucose tolerance tests

Lean rats received Rb1 (10 mg/kg, i.p.) or saline daily for 5 days, and after Day 4, the rats were fasted overnight. Four hours after the final injection on Day 5, an intraperitoneal glucose tolerance test (ipGTT) was conducted as described previously (Xiong et al. 2010). Briefly, the rats received i.p. glucose (1 g/kg, at 1100 h), and blood samples were taken from the tail vein at 0 (fasting), 15, 30, 60, and 120 min after glucose administration. Glucose was assessed with a glucometer (Freestyle; Abbott Diabetes Care, Alameda, CA), and the area under the 120-min glucose curve was calculated by the trapezoidal rule. Plasma insulin levels were measured with an ultrasensitive rat insulin ELISA kit (Cat. #: 90060, Crystal Chem Inc., Downers Grove, IL).

### Insulin tolerance tests

After a 7-day washout, the same rats received Rb1 (10 mg/kg per day, i.p.) or saline for an additional 5 days. On Day 5, an insulin tolerance test (ITT) was performed after a 4-h fast in the mid-light cycle by an injection of insulin (0.5 unit/kg, i.p.), with glucose levels determined at 0 (fasting), 15, 30, 45, 60, 90, 120, and 150 min from blood samples taken from the tail vein.

### Euglycemic-hyperinsulinemic clamp

Rats were divided into three groups: one group was fed LFD (*n* = 7), and the other two groups were fed HFD for 4 weeks (*n* = 9/group). The rats were then anesthetized with isoflurane, and indwelling catheters were inserted into the right internal jugular vein and the left carotid artery, respectively (Obici et al. 2002a, b). The venous catheter was extended to the level of the right atrium, and the arterial catheter was advanced to the level of the aortic arch. The catheters were tunneled subcutaneously, exteriorized at the back of the neck, and filled with heparinized saline. The jugular and carotid catheters were used for infusion and blood sampling, respectively. While remaining on their diets, after 2-day recovery from surgery, the rats received Rb1 (10 mg/kg, i.p.) or saline daily for 5 days. Food intake and body weight were monitored daily.

On Day 5 of Rb1 administration, hyperinsulinemic-euglycemic clamps were performed in conscious rats that were fasted for 4 h, as described in Figure 2C (Rossetti et al. 1987, 1997; Obici et al. 2002a,b). Briefly, a primed-continuous infusion of HPLC-purified [3-^3^H]-glucose (40 *μ*Ci bolus, 0.4 *μ*Ci/min) was administered for the duration of the study (Liu et al. 1998; Obici et al. 2002a,b). Two hours after the basal period, a primed continuous infusion of regular human insulin (60 mU/kg bolus followed by 1.8 mU/kg per min) was begun, and the start time was counted as *t* = 0 min. A variable infusion of a 25% glucose solution was started at time 0 and periodically adjusted to clamp plasma glucose concentration at approximately 7.5 mmol/L for the remainder of the experiment. To estimate insulin-stimulated glucose transport activity in individual tissues, 2-[^14^C]DG was administered as a bolus (25 *μ*Ci) at 45 min in HFD-fed rats before the end of clamps. Serial blood samples were obtained throughout the basal and clamp periods for determination of plasma glucose, insulin, [^3^H]glucose, ^3^H_2_O, and 2-[^14^C]DG concentrations for calculation of glucose turnover rates as described previously (German et al. 2009). All blood samples were immediately centrifuged, and plasma was stored at –80°C for subsequent analysis. To prevent volume depletion and anemia, a solution (1:1, vol/vol) of red blood cells collected during the clamp and heparinized saline was infused periodically. After terminal blood sampling at 120 min, the animals were promptly euthanized and liver and skeletal muscles (soleus muscle) were collected. Each tissue was frozen immediately using liquid N_2_-cooled aluminum blocks and stored at –80°C for subsequent metabolic analysis.

### In vivo glucose flux analysis

Plasma glucose concentration was analyzed during clamps using 10 *μ*L plasma with a glucose oxidase method on a GM7 Analyzer (Analox Instruments Limited, London, UK). Plasma insulin concentration was measured with an ultrasensitive rat insulin ELISA kit. For the determination of plasma [3-^3^H]glucose and 2-[^14^C]DG concentrations, plasma was deproteinized with ZnSO_4_ and Ba(OH)_2_, dried to remove ^3^H_2_O, resuspended in water, and counted in scintillation fluid by LS 6500 multipurpose scintillation counter (Beckman Coulter, Indianapolis, IN) on dual channels for separation of ^3^H and ^14^C. The plasma concentration of ^3^H_2_O was determined by the difference between ^3^H counts with and without drying.

Under steady-state conditions for plasma glucose concentration, the rate of glucose disappearance (*R*_d_) equals the rate of glucose appearance (*R*_a_). The latter was calculated as the ratio of the rate of infusion of [3-^3^H]glucose (disintegrations per minute, dpm) and the steady-state plasma [^3^H]glucose-specific activity (dpm/mg). When exogenous glucose was given, the rate of endogenous glucose production was calculated as the difference between *R*_a_ and the glucose infusion rate (Obici et al. 2001).

### Glucose uptake

For glucose uptake analysis, skeletal muscle (60–100 mg) was added into 250 *μ*L of 1N NaOH and incubated at 80°C for 30 min to digest the tissues. The solutions were neutralized with 250 *μ*L of 1N HCl, and then centrifuged at 14,000 rpm for 15 min. An aliquot of the supernatant was precipitated by HClO_4_ and quantified by liquid scintillation counting to determine total tissue values (dpm) for the sum of 2-[^14^C]DG and 2-[^14^C]deoxyglucose phosphate (2-[^14^C]DGP). Another aliquot was deproteinized with 0.3N zinc sulfate (ZnSO_4_) and 0.3N barium hydroxide [Ba(OH)_2_] to precipitate 2-[^14^C]DG6P and quantify 2-[^14^C]DG in the supernatant. The value for the 2-[^14^C]DG in the supernatant (dpm) was subtracted from the total tissue 2-[^14^C]DG and 2-[^14^C]DGP (dpm) to calculate the glucose uptake rate as indicated by the skeletal muscle 2-[^14^C]DGP accumulation (Halseth et al. 1999).

### Analysis of the rate of glycogen synthesis

To determine the rate of glycogen synthesis, liver and muscle tissue (60˜100 mg) were rinsed with cold phosphate-buffered saline (PBS) and solubilized by incubating with 1 mol/L KOH (0.5 ml) at 80°C for 30 min. After centrifugation, the supernatant was transferred to a new tube, 95% ethanol (550 *μ*L) was added and stored at –20°C overnight. After centrifugation, the pellet was washed with ice-cold 95% ethanol and resuspended with 300 *μ*L ddH_2_O. Part of the solution (150 *μ*L) was transferred in duplicate to scintillation vials, and ^14^C-radioactivity was counted. The activity per tissue mass was then normalized to the integral of the plasma specific activity of the 2-[^14^C]DG over the time of exposure, to yield a rate of incorporation into glycogen (Huang and Veech 1988).

### Quantitative real-time RT-PCR analysis

Total RNA was extracted from liver samples with an RNAqueous-Micro Kit (Life Technologies, Grand Island, NY) and treated with DNase I before cDNA synthesis (Shen et al. 2014). The DNase I-treated total RNA (1 *μ*g) was reverse-transcribed to first-strand cDNA following the manufacturer’s instructions (GE Healthcare Bio-Sciences, Piscataway, NJ). Quantitative real-time PCR was performed in a 20 *μ*L final reaction volume with TaqMan Master mix using an StepOnePlus^–^ system (Life Technologies, Carlsbad, CA). Real-time PCR conditions were as follows: 95°C for 3 min for one cycle followed by 38 cycles of 95°C for 20 sec and 60°C for 20 sec. Threshold cycle readings for each of the unknown samples were then used, and the results were analyzed using the ΔΔCT method described previously (Xiong et al. 2010). Rat 45S rRNA levels from each sample were used as internal controls to normalize the mRNA levels. The predesigned TaqMan® Gene Expression Assay IDs (Life Technologies, Carlsbad, CA) were as follows: rat phosphoenolpyruvate carboxykinase (Pepck): Rn01529014_m1; rat glucose-6-phosphatase (glu-6-pase): Rn00598561_m1; and rat 45 S: Rn03928990_g1.

### Immunoblotting analysis

Total proteins were extracted from muscle using the method described previously (Treebak et al. 2010; Shen et al. 2013). The extracted proteins (30 *μ*g) were separated by 4–15% SDS-PAGE gradient gels (Bio-Rad Life Science, Hercules, CA) and then electrotransferred to polyvinylidene fluoride membranes (Millipore, Bedford, MA). After incubating in blocking buffer, the membranes were incubated with an antiphospho-AMPK*α* (Thr 172) antibody (1:1000 dilution) overnight at 4°C. After washing with PBS, the membranes were incubated with horseradish peroxidase-conjugated secondary antibody (1:5000 dilution; Dako, Carpinteria, CA). The amount of immune complexes was quantitated using an enhanced chemiluminescence detection system (EMD Millipore, Billerica, MA). The reacted membranes were exposed to X-ray film (Kodak Scientific, Rochester, NY). Membranes were then stripped and reblotted with anti-AMPK*α* (1:1000 dilution) to verify equal loading of protein in each lane. The same procedure was used for the measurements of pACC and ACC, pIRS-1 and IRS-1, pAkt and Akt, pTBC1D4 and TBC1D4, and GLUT4 and actin levels. Film density, measured as transmittance, was expressed as volume-adjusted optical density. The amount of each protein was normalized to the respective individual density values reflecting protein level of AMPK*α* and was expressed as a ratio.

### Statistical analyses

All data are presented as mean ± standard error (SE). Data were analyzed using parametric statistics (SigmaPlot version 12.0). Differences among more than two groups were determined using one-way analysis of variance followed by the Tukey test for comparison between treatments. Student’s *t*-test was used to compare the effects between Rb1 and vehicle treatments. *P*s < 0.05 were considered statistically significant.

## Results

### Rb1 improves glucose tolerance and insulin sensitivity in lean rats

**Figure 1:**
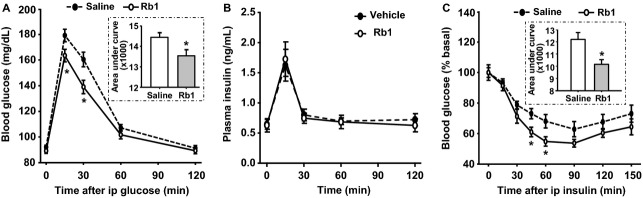
Administration of Rb1 improves glucose tolerance and insulin sensitivity in lean rats. Blood glucose (A) and plasma insulin (B) levels during intraperitoneal glucose tolerance test (ipGTT), and blood glucose (C) during insulin tolerance test (ITT) in rats after Rb1 (10 mg/kg, i.p.) or vehicle (saline) treatment for 5 days. Data are mean ± SE; *n *=* *8. *P <* *0.05, compared with saline controls.

To determine whether the effect of Rb1 on insulin sensitivity is limited to obese rats, either saline or Rb1 (10 mg/kg per day) was administered i.p. for 5 days to chow-fed lean rats. No significant differences were found between the vehicle and Rb1 groups in daily food intake (24.5 ± 2.6 g vs. 22.4 ± 1.8 g) and the changes of body weight (14.6 ± 1.4 g vs. 11.3 ± 2.2 g) during the 5 days of treatment. Fasting blood glucose was comparable between the two groups over the 5 days. During the ipGTT, Rb1-treated rats had significantly lower blood glucose at the 15 and 30-min time points, as well as a significantly reduced area under the curve (Fig. 1A), compared to saline-treated rats. Thus, glucose tolerance was increased in Rb1-treated rats. Because there were no differences in plasma insulin at any time point between Rb1- and vehicle-treated rats (Fig. 1B), this implies that Rb1 increases insulin sensitivity.

During the ipITT, Rb1-treated rats had significantly lower blood glucose at 45 and 60 min, as well as reduced area under the curve (Fig. 1C), again indicating that Rb1 increased insulin sensitivity in lean rats fed standard rodent chow.

### Rb1 enhances insulin-stimulated whole-body glucose flux

**Figure 2:**
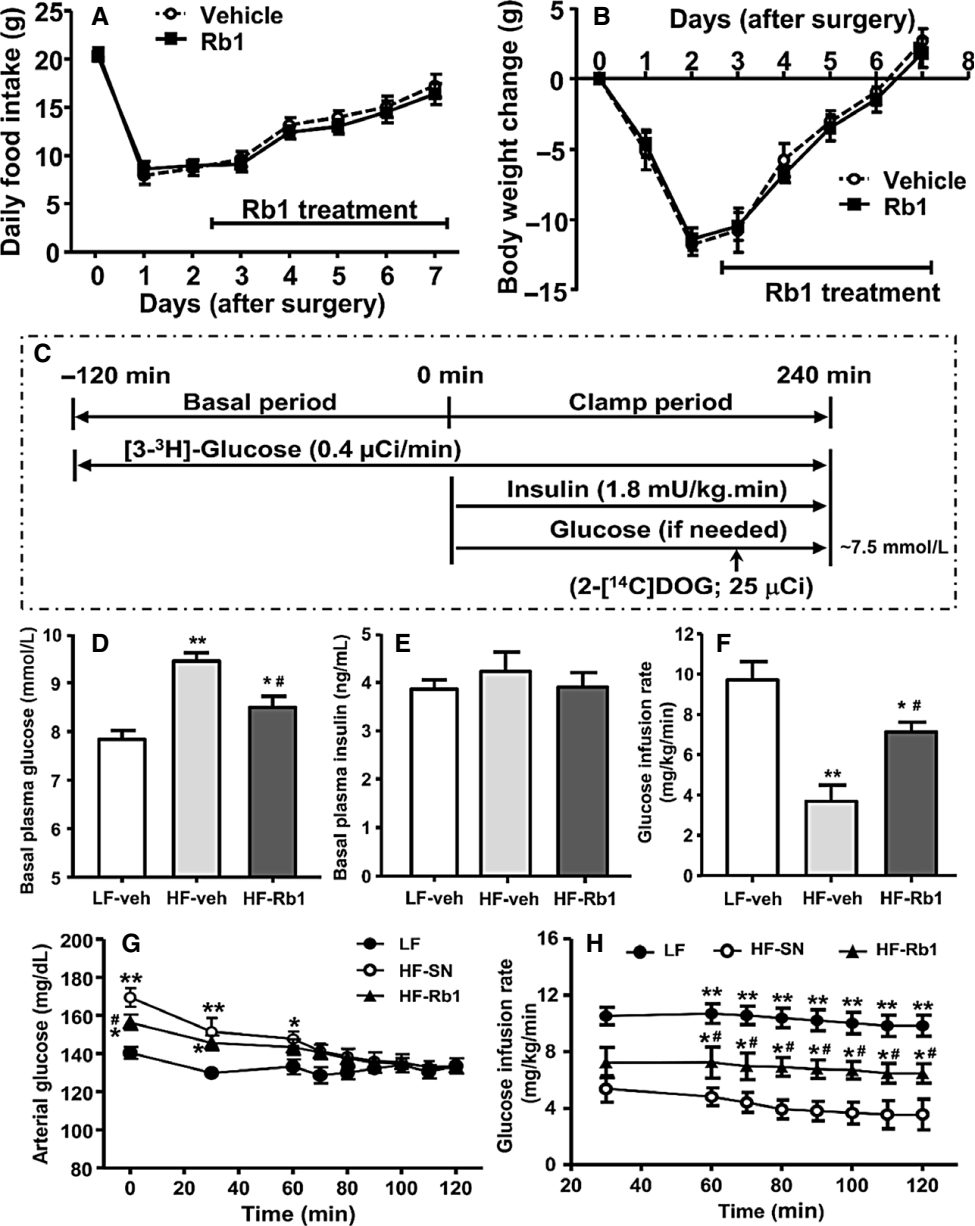
Rb1 treatment for 5 days did not significantly affect daily food intake (A) or body weight change (B) in high-fat diet (HFD)-fed rats. Schematic representation of the pancreatic clamp (C). Maintenance on a HFD caused a significant increase in fasting glucose, and this was attenuated in Rb1-treated HFD-fed rats, compared to that in low-fat diet (LFD)-fed rats (D), although steady-state insulin levels were comparable among the three groups (E). Steady-state glucose infusion rate (F) obtained from averaged rates over 90–120 min of euglycemic-hyperinsulinemic clamp in the three groups of rats. Arterial glucose levels (G) and glucose infusion rate (H) were measured during the euglycemic-hyperinsulinemic clamp in these three groups of rats. Mean±SE, *n* = 7–9. ^*^P <* *0.05 and ^**^P <* *0.01 vs. vehicle (veh)-treated rats on LFD; and ^*#*^*P *<* *0.05 vs. vehicle-treated rats on HFD.

To directly examine the effect of Rb1 on insulin sensitivity, euglycemic-hyperinsulinemic clamps were performed on LFD- and HFD-fed rats on Day 5 of consecutive i.p. administration of either vehicle or Rb1 (10 mg/kg per day). No significant differences in daily food intake or body weight change were found in Rb1-treated rats compared with that of vehicle control (Fig. 2A and B). As depicted in Figure 2C, a euglycemic-hyperinsulinemic clamp protocol was conducted in conscious rats fasted for 4 h. In basal conditions, fasting plasma glucose was increased in HFD obese rats as compared to chow-fed lean rats, while Rb1 treatment reduced plasma glucose levels (Fig. 2D). Insulin levels were comparable among all three groups (Fig. 2E).

During the clamp procedure, arterial glucose concentrations were matched among the three groups (Fig. 2G). The glucose infusion rate, required to maintain euglycemia, was decreased in vehicle-treated HFD rats compared to that of vehicle-treated LFD-fed controls (Fig. 2H), indicating that HFD rats had developed insulin resistance. HFD rats receiving Rb1 had significantly increased glucose infusion rate (an increase of 98%), compared with HFD vehicle-treated rats (Fig. 2H and F), indicating that Rb1 treatment had improved insulin sensitivity in HFD-fed rats.

### Rb1 improves insulin’s effect on hepatic glucose production

**Figure 3:**
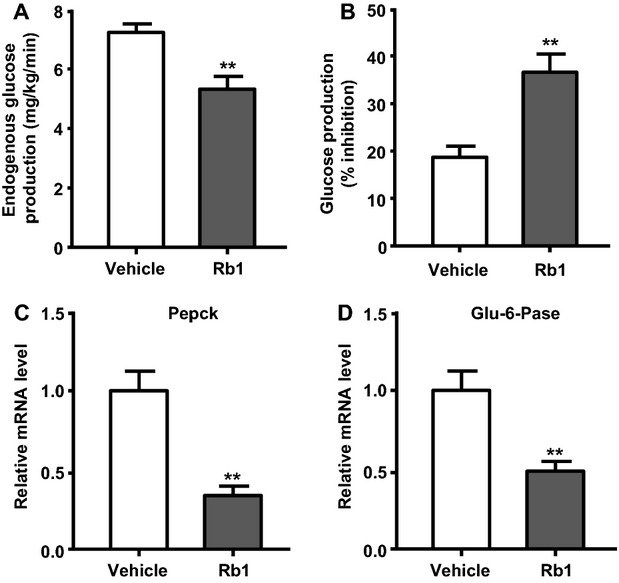
Rb1 significantly enhances insulin-mediated inhibition of endogenous glucose production and suppression of hepatic gluconeogenic gene expression. Rate of endogenous glucose production during the clamp period (A), and percent suppression of hepatic glucose production (B). For gene expression measurements, liver RNA was extracted from the rats treated with Rb1 or vehicle, and phosphoenolpyruvate carboxykinase (Pepck) (C) and glucose-6-phosphatase (glu-6-pase) (D) mRNA levels were quantitated by real-time PCR and normalized to rat 45S. Mean ± SE, *n* = 7–9. *^*^P <* *0.01 vs. vehicle controls.

One of insulin’s major actions is to diminish the rate of endogenous glucose production by the liver. The increased glucose infusion rate was accompanied by an Rb1-enhanced insulin action to lower the rate of glucose production (measured as the difference between glucose appearance [*R*_a_] and glucose infusion rate), relative to rats receiving vehicle (Fig. 3A). The effect of Rb1 on hepatic glucose production is also expressed as percentage of inhibition from basal levels (Fig. 3B). To gain insight into the molecular mechanism underlying the insulin-sensitizing effects of Rb1, we measured the mRNA levels of two key gluconeogenic enzymes, Pepck and glu-6-pase, in liver samples obtained after the completion of the clamp. Both Pepck and glu-6-pase mRNA levels were significantly reduced (by 70.6% and 50.5%, respectively) in animals receiving Rb1, compared to that in control rats (Fig. 3C and D). Thus, Rb1 reduced hepatic gluconeogenesis, in part, by suppressing the expression of these key gluconeogenic genes.

### Rb1 increases glucose uptake

**Figure 4:**
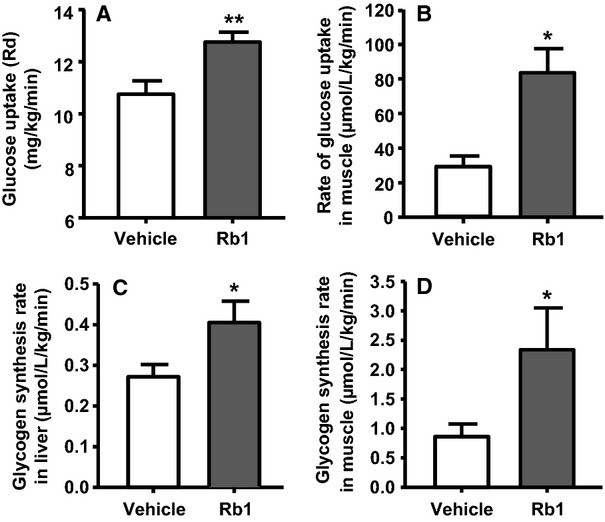
Rb1 treatment significantly increases the rate of whole-body glucose uptake (A) in rats fed high-fat diet (HFD) for 4 weeks. The increased whole-body glucose uptake rate was accompanied by greater glucose uptake in skeletal muscle (B) of Rb1-treated rats, compared to those in vehicle-treated HFD-fed rats. Additionally, Rb1 significantly increases the rate of glycogen synthesis in liver (C) and skeletal muscle (D) compared with vehicle. Mean ± SE, *n* = 7–9. ^*^P <* *0.05 vs. vehicle-treated controls.

Another important action of insulin is to stimulate the uptake of glucose by peripheral tissues. Consistent with that, the rate of whole-body glucose uptake, also called the rate of glucose disposal (*R*_d_), was significantly increased in Rb1-treated HFD-fed rats, compared with that in vehicle controls (Fig. 4A). Figure 4B demonstrates that Rb1 significantly increased glucose uptake in skeletal muscle, compared to vehicle treatment.

### Rb1 increases the rate of glycogen synthesis

The rates of glycogen synthesis in liver (Fig. 4C) and skeletal muscle (Fig. 4D) of Rb1-treated HFD-fed rats were significantly increased, compared to those in vehicle-treated controls. This observation implies that Rb1-induced glucose uptake by those tissues might enhance the rate of glycogen synthesis and storage in HFD-fed rats.

### Rb1 does not change plasma lipid profile

Analysis of plasma samples taken after the completion of the clamp revealed no significant differences in plasma concentrations of triglyceride, cholesterol, phospholipid, or nonesterified fatty acids between the Rb1 and vehicle groups (Table 1). These data suggest that the improved insulin sensitivity induced by Rb1 in rats fed a HFD is independent of any changes in the lipid profile.

**Table 1:** Lipid profiles in high-fat diet (HFD)-fed rats with or without Rb1 treatment

	Vehicle	Rb1
Triglyceride (mg/dL)	117.6 ± 18.72	81.3 ± 17.39
Cholesterol (mg/dL)	55.3 ± 2.10	50.8 ± 3.09
Phospholipids (mg/dL)	0.153 ± 0.012	0.155 ± 0.009
Nonesterified fatty acids (mEq/L)	1.13 ± 0.255	1.24 ± 0.154

Data are presented as mean ± SE. No significant differences occurred between these two groups.

### Rb1 does neither enhance proximal insulin signaling nor the activation of Akt in muscle

**Figure 5:**
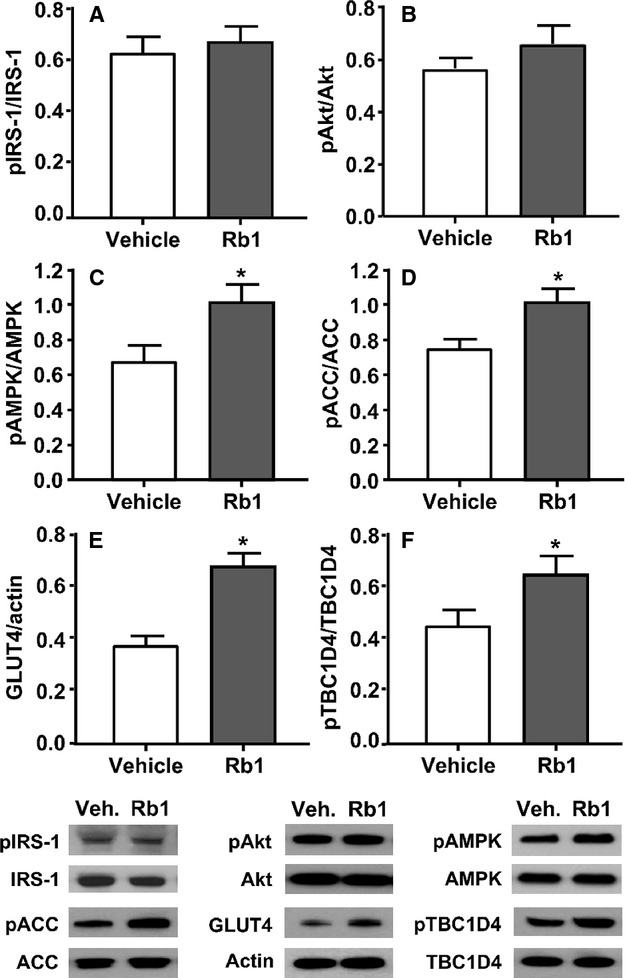
Effects of Rb1 on insulin and AMPK signaling pathways in the skeletal muscle of rats fed high-fat diet (HFD) for 4 weeks. Quantitative analyses for (A) protein levels of phosphorylated IRS-1 (pIRS-1) and total IRS-1 (IRS-1); (B) phosphorylated Akt (pAkt) and total Akt (Akt); (C) phosphorylated AMPK*α* (pAMPK*α*) and total AMPK (AMPK*α*); (D) phosphorylated ACC (pACC) and total ACC (ACC); (E) protein levels of GLUT4 versus actin; (F) phosphorylated TCB1D4 (pTCB1D4) and total TCB1D4 (TCB1D4); and (G) representative western blot images from Rb1- and vehicle-treated rats. AU: arbitrary units. Mean ± SE, *n* = 7–9, ^*^P <* *0.05 vs. vehicle-fed controls. IRS, insulin receptor substrate; ACC, acetyl-CoA carboxylase.

To identify potential molecular mechanisms underlying the Rb1-induced increase of glucose uptake in skeletal muscle, we analyzed the fully insulinized skeletal muscle harvested at the termination of the glucose-clamp study. The tissue was homogenized and subjected to immunoblotting, after which we measured the levels of pIRS-1 and pAkt, to determine the signaling efficacy through the IRS-1/PI3K/Akt axis. As depicted in Figure 5A and B, Rb1 did not significantly alter the activation of either IRS or Akt in muscle of HFD-fed rats compared with vehicle control.

### Rb1 increases AMPK*α* phosphorylation and GLUT4 levels in muscle

Rb1 could potentially increase glucose uptake in skeletal muscle by regulating the activation of AMPK*α*. To investigate this, we measured the phosphorylation of AMPK*α*. Rb1 significantly increased phosphorylation of AMPK*α* in skeletal muscle, compared to vehicle, whereas the total amount of AMPK*α* protein was not significantly changed (Fig. 5C). Consistent with this, Rb1 treatment also elevated the level of phosphorylated ACC (Fig. 5D). Additionally, the levels of GLUT4 in skeletal muscle were significantly increased in Rb1-treated rats compared to levels in vehicle controls (Fig. 5E).

### Rb1 enhances TBC1D4 signaling

We evaluated TBC1D4 phosphorylation status because this protein is a convergence point for Akt and AMPK signaling in skeletal muscle (Kramer et al. 2006; Treebak et al. 2010). While TBC1D4 is phosphorylated at multiple sites, the phosphorylation of TBC1D4 Thr^649^ is critical for insulin- and AMPK-stimulated glucose uptake (Chen et al. 2011; Kjøbsted et al. 2014). An increased effect of insulin on TBC1D4 Thr^649^ phosphorylation was found in the muscle of Rb1-treated rats, compared with that in vehicle-control rats (Fig. 5F).

## Discussion

Although ginseng has been reported to ameliorate hyperglycemia in basic (Kimura et al. 1981; Attele et al. 2002; Franz et al. 2003; Yun et al. 2004) and clinical studies (Vuksan et al. 2000a, 2001b), the effective component(s) and molecular mechanisms are largely unknown. Our previous studies demonstrated that chronic treatment with Rb1 significantly reduces fasting glucose and improves glucose tolerance in HFD-induced obese rats without increasing plasma insulin (Xiong et al. 2010). Since the effects were greater than those observed in pair-fed rats, the data suggest a direct effect of Rb1 on glucose homeostasis. This possibility was confirmed in the present study. In normal lean rats fed standard rodent chow, 5-day treatment with Rb1 (10 mg/kg, i.p.) significantly improved glucose tolerance and enhanced insulin sensitivity. Notably, those effects were not accompanied by reduced food intake or changed body weight.

To identify the mechanisms of how Rb1 enhances insulin sensitivity, euglycemic-hyperinsulinemic clamps were performed on HFD-fed rats on Day 5 of consecutive i.p. administration of either vehicle or Rb1 (10 mg/kg per day). No significant differences in daily food intake or body weight change were found in Rb1-treated rats compared with that of vehicle controls during the short-term (5 days) consumption of a HFD. The design of this experiment differs from that of our previous studies (Xiong et al. 2010), where the rats were maintained on HFD for more than 13 weeks. These observations suggest that Rb1 protects against metabolic disorders via mechanisms that are independent of a change in food intake and body weight. Through the clamps, we demonstrated that Rb1 significantly increased the glucose infusion rate required to maintain euglycemia and enhanced whole-body glucose disposal in HFD-fed rats, implying that Rb1 improves whole-body insulin sensitivity. Consistent with this, Rb1 significantly enhanced insulin-induced inhibition of hepatic gluconeogenesis and suppression of Pepck and glu-6-pase gene expression. Additionally, glucose uptake in muscle and glucose incorporation into glycogen in both liver and muscle were significantly increased. Molecular studies revealed that Rb1 treatment did not significantly alter either proximal insulin signaling or the activation of Akt in skeletal muscle, whereas it significantly increased the phosphorylation of AMPK and TBC1D4, a downstream component of the AMPK pathway. These data suggest that Rb1 improves insulin sensitivity by activating AMPK in skeletal muscle.

The observation that short-term (5 days) administration of Rb1 increased insulin sensitivity in lean chow-fed rats is consistent with a previous report in healthy, nondiabetic humans (Vuksan et al. 2000b, 2001a). For example, in a random crossover design, American ginseng (3 g) that was taken 40 min before a 25 g oral glucose challenge reduced plasma glucose and resulted in a significantly lower area under the curve, when compared to the placebo (Vuksan et al. 2000b). While it is impossible to know the level of Rb1 that existed in those ginseng extracts, the implication is that Rb1, being the most abundant ginsenoside in ginseng, might have made an important contribution to the improved glucose tolerance in those nondiabetic individuals.

T2DM is a progressive disease, characterized by insulin resistance and impaired insulin secretion, leading to chronic hyperglycemia (Olefsky and Glass 2010). Insulin resistance, a central component defining the metabolic syndrome, is marked by the impaired ability of insulin to inhibit glucose output from the liver and to promote glucose uptake into peripheral tissues, especially skeletal muscle (Olefsky and Glass 2010), and is frequently found in individuals with impaired glucose tolerance (Flier 2004). Evidence from previous studies has suggested that Rb1 can reverse the metabolic disorders of T2DM (Xiong et al. 2010), but until now, the mechanism for this effect has remained unclear.

Hyperglycemia is particularly related to hepatic glucose synthesis. Excessive hepatic gluconeogenesis is responsible for the high blood glucose and can lead to insulin resistance (Cheng et al. 2006). Thus, suppression of gluconeogenesis in the liver is suggested as an intervention for T2DM. Insulin inhibits gluconeogenesis by suppressing the expression of both of Pepck and glu-6-pase, which are key enzymes of hepatic gluconeogenesis (Yoon et al. 2001). In the present experiment, we examined the expression of the genes encoding the hepatic gluconeogenic enzymes Pepck and glu-6-pase in Rb1- and vehicle-treated rats, and found that Rb1 significantly enhanced the action of insulin in inhibiting hepatic glucose production, and the reduced glucose production was accompanied by suppressed expression of Pepck and glu-6-pase genes (Fig. 3).

Stimulation of glucose uptake in skeletal muscle is one of the most important processes regulated by insulin (Taniguchi et al. 2006). Skeletal muscle has been reported to account for 70–75% of insulin-stimulated glucose disposal during hyperinsulinemic clamps and, therefore, represents a principal tissue affecting whole-body glucose homeostasis (DeFronzo et al. 1981; Shulman et al. 1990). In the present study, we found that the rate of whole-body glucose uptake, and in particular uptake in skeletal muscle, was significantly increased in Rb1-treated HFD-fed rats, compared with the levels in vehicle controls (Fig. 4A and B). These observations are supported by previous reports that Rb1 stimulated glucose uptake in C2C12 myotubes (Tabandeh et al. 2015) and cardiomyocytes (Kong et al. 2009). Additionally, Rb1 increased basal and insulin-mediated glucose uptake in 3T3-L1 adipocytes, and this was associated with increased gene expression of GLUT4, as well as GLUT4 translocation to the cell surface (Shang et al. 2008).

Other than enhanced glucose uptake in muscle, the rates of glycogen synthesis in liver (Fig. 4C) and skeletal muscle (Fig. 4D) of Rb1-treated HFD-fed rats were significantly increased, compared to those in vehicle-treated controls. This observation implies that Rb1-induced glucose uptake by those tissues might enhance the rate of glycogen synthesis and storage in HFD-fed rats.

Insulin stimulates glucose uptake into skeletal muscle through an increase of GLUT4 translocation from intracellular storage vesicles to the plasma membrane and transverse tubules (Lauritzen et al. 2008). Insulin initiates its effect by binding to the insulin receptor, followed by receptor autophosphorylation, which in turns induces a cascade of phosphorylation and protein–protein interactions leading to increased translocation of GLUT4 (Shepherd 2005). Insulin activates insulin receptor tyrosine kinase activity that increases the tyrosine phosphorylation of insulin receptor substrate (IRS) proteins, which in turn recruit and activate phosphatidylinositol-3-kinase (PI3K). Activation of PI3K catalyzes the formation of phosphatidylinositol-3,4,5-trisphosphate (PIP3), which recruits both Phosphoinositide-dependent kinase-1 (PDK1) and Akt to the phospholipid, and subsequently allows Akt to be activated through phosphorylation by PDK1 (Alessi and Cohen 1998). Several lines of evidence have identified the critical role of Akt phosphorylation and activation in the regulation of insulin-stimulated glucose uptake (Larance et al. 2008). However, the enhanced glucose uptake in muscle by Rb1 treatment appears to be independent of changes in proximal insulin signaling because our current findings demonstrate that Rb1 treatment did not affect either IRS or Akt phosphorylation in rat skeletal muscle (Fig. 5A and B).

Exercise and muscle contractions potently stimulate glucose uptake, but in a manner independent of insulin (Goodyear and Kahn 1998; Kennedy et al. 1999). Compelling evidence suggests that AMPK could potentiate increases in glucose transport in response to exercise (Jessen and Goodyear 2005). AMPK is a key sensor of cellular energy status and has been implicated in the control of glucose homeostasis (Kahn et al. 2005). AMPK consists of a heterotrimeric complex containing catalytic subunit (*α*1/*α*2) and regulatory subunits (*β*1/*β*2 and *γ*1/*γ*2/*γ*3). Activation of AMPK by phosphorylation of its *α*-subunit at a threonine residue (Thr^172^) leads to the phosphorylation and regulation of a number of downstream targets involved in glucose metabolism (Hardie 2013). A growing number of in vivo and in vitro studies have indicated that the activated AMPK signaling pathway induces an increase in glucose uptake in peripheral tissues, especially muscle, by stimulating GLUT4 gene expression and/or translocation to the plasma membrane (Kurth-Kraczek et al. 1999). This insulin-independent mechanism is critical for the maintenance of glucose homeostasis. Indeed, the drug metformin, an oral hypoglycemic agent for the treatment of T2DM, appears to improve insulin sensitivity mainly via the activation of AMPK in muscle (Fryer et al. 2002; Musi et al. 2002).

We hypothesized that Rb1-enhanced glucose uptake could result from increased AMPK activation in muscle. To test this, we determined the phosphorylation of AMPK*α* in HFD-fed rats treated with/without Rb1. Concomitant with our hypothesis, 5-day treatment with Rb1 significantly enhanced AMPK activation in skeletal muscle (Fig. 5C), which was confirmed by elevated level of phosphorylated ACC, a substrate of active AMPK (Fig. 5D), suggesting that targeting AMPK signaling is an important aspect of Rb1’s antihyperglycemic function. Consistent with the changes in AMPK activation, the levels of GLUT4 in skeletal muscle were significantly increased in Rb1-treated rats compared to vehicle controls (Fig. 5E).

TBC1D4 is involved in glucose transport in skeletal muscle (Kramer et al. 2006; Chen et al. 2011) and is regulated via phosphorylation by both Akt and AMPK (Treebak et al. 2010; Kjøbsted et al. 2014). Since insulin (Akt) and AMPK signaling pathways converge on TBC1D4, the enhanced activation of AMPK by Rb1 could explain how Rb1 increases glucose uptake in fully insulinized skeletal muscle. Supporting this concept, TBC1D4 phosphorylation was elevated in skeletal muscle of rats treated with Rb1 relative to that in vehicle controls, concomitant with increased AMPK activation and stimulated glucose uptake after Rb1 administration. The data suggest that, like exercise and metformin, Rb1 increases AMPK activity in skeletal muscle, and the increased phosphorylation of TBC1D4 facilitates the effect of Rb1-induced AMPK activation to enhance glucose uptake in response to insulin. On the other hand, unlike metformin (Bailey and Turner 1996), we previously reported that chronic administration of Rb1 to HFD-induced obese rats decreases food intake and fat mass. Thus, these preclinical studies demonstrate that Rb1 can potentially become an effective treatment for obesity, insulin resistance, and T2DM.

In conclusion, our findings present direct evidence supporting a novel and critical role of Rb1 in the regulation of glucose metabolism. During consecutive 5-day treatment, Rb1 significantly reduced hepatic glucose production, and this effect was likely mediated by suppressing the expression of Pepck and glu-6-pase. Additionally, Rb1 significantly increased glucose uptake in skeletal muscle and glucose incorporation into glycogen in the liver and skeletal muscles. These effects seem independent of activation of proximal insulin signaling because Rb1 did not change the phosphorylation of IRS-1. However, the increased phosphorylation of TBC1D4, a downstream target of AMPK signaling, appears to be a key part of the mechanism for Rb1-stimulated glucose uptake in skeletal muscle. These findings contribute a new understanding as to how Rb1 enhances insulin sensitivity and affects glucose metabolism, and provide strong evidence for further evaluation of the potential therapeutic role of Rb1 in hyperglycemia or diabetes.
